# Disinfectants in a Hemodialysis Setting: Antifungal Activity Against *Aspergillus* and *Fusarium* Planktonic and Biofilm Cells and the Effect of Commercial Peracetic Acid Residual in Mice

**DOI:** 10.3389/fcimb.2021.663741

**Published:** 2021-04-29

**Authors:** Leonardo G. Lopes, Larissa A. Csonka, Jessica A. Souza Castellane, Alef Winter Oliveira, Sílvio de Almeida-Júnior, Ricardo Andrade Furtado, Cibele Tararam, Larissa Ortolan Levy, Leandro Zuccolotto Crivellenti, Maria Luiza Moretti, Maria José S. Mendes Giannini, Regina H. Pires

**Affiliations:** ^1^ Postgraduate Program in Health Promotion, University of Franca, Franca, Brazil; ^2^ Postgraduate Program in Animal Sciences, University of Franca, Franca, Brazil; ^3^ Faculty of Medical Sciences, University of Campinas, Campinas, Brazil; ^4^ Faculty of Veterinary Medicine, Federal University of Uberlandia, Uberlandia, Brazil; ^5^ School of Pharmaceutical Sciences, São Paulo State University (UNESP), Araraquara, Brazil

**Keywords:** *Aspergillus*, *Fusarium*, biofilm, disinfectant, mice

## Abstract

*Aspergillus* and *Fusarium* cause a broad spectrum of infections in humans, mainly in immunocompromised patients. Among these, patients undergoing hemodialysis are highly susceptible to infections, requiring a constant and adequate environmental disinfection program. Nevertheless, monitoring the residual disinfectants can contribute to the morbidity and mortality reduction in these patients. Here, we evaluated the susceptibility of *Aspergillus* spp. (n=19) and *Fusarium* spp. (n=13) environmental isolates against disinfectants (acetic acid, citric acid, peracetic acid, sodium hypochlorite, and sodium metabisulphite) at different concentrations and time exposures. Also, we investigated the *in vivo* toxicity of the peracetic acid residual concentration in mice. *Fusarium* isolates were identified by *F. equiseti*, *F. oxysporum* and *F. solani* while *Aspergillus* presented clinically relevant species (*A. fumigatus*, *A. niger* and *A. terreus*) and environmental ones. Against planktonic cells, only two disinfectants (acetic acid and sodium hypochlorite) showed a fungicidal effect on *Fusarium* spp., while only one (sodium hypochlorite) was effective against *Aspergillus* spp. Both fungi formed robust *in vitro* biofilms with large amounts of the extracellular matrix, as evidenced by electron micrographs. Exposure of fungal biofilms to disinfectants showed sensitivity to three (acetic, citric, and peracetic acids), although the concentrations and times of exposure varied according to the fungal genus. Mice exposure to the residual dose of peracetic acid during 60 weeks showed anatomopathological, hematological, and biochemical changes. The implementation of news control measures and those that already exist can help reduce infections, the second cause of death and morbidity in these patients, besides providing safety and well-being to them, a priority of any quality health program.

## Introduction

Chronic renal patients, due to abnormalities in their immune systems, such as T cell deficiency, accumulation of pro-inflammatory cytokines, oxidative stress, among others, are more susceptible to infections, which are the second most common cause of morbidity and mortality in these patients (Kato et al., 2008; Chang et al., 2020). Among these, invasive fungal infections present high rates among immunocompromised patients, especially those undergoing hemodialysis (Ghandi et al., 2005).

Fungi can be contaminants in the hemodialysis system over the years (Arvanitidou et al., 2000; Varo et al., 2007; Pires-Gonçalves et al., 2008; Figel et al., 2013; Schiavano et al., 2014; Abbass et al., 2018; Nehmatullah, 2020), although monitoring such pathogens, whether in planktonic or biofilm growth, has not received much attention from regulatory agencies.

*Aspergillus* spp., *Fusarium* spp., and *Candida* spp. have been highlighted as fungi contaminant, mainly from water used in hemodialysis (Arvanitidou et al., 2000; Pires-Gonçalves et al., 2008; Pires et al., 2011). *Aspergillus*, a filamentous fungus, are saprobic organisms found in the environment and were considered an important opportunistic agent in clinical practice (Hsieh et al., 2017). Factors such as i) reduced conidia size, which facilitates inhalation of conidial forms; ii) temperature found in the upper airways is practically the same as the fungus develops in nature; iii) great adherence capacity and biofilm formation, contribute to the development of pathologies associated with *Aspergillus* in immunocompromised patients (Müller et al., 2011; Lass-Flörl et al., 2013; Beauvais and Latgé, 2015).

*Fusarium* has been found in the hospital environment on the water distribution system, and its conidia can be aerosolized by opening taps, showers, or flush toilets (Anaissie et al., 2001; Muhammed et al., 2011; Sautour et al., 2012; Moretti et al., 2018). This fungal adaptation to the hospital aquatic environments has allowed correlating *Fusarium* isolates obtained from the water with isolates obtained from hospitalized patients (O’Donnell et al., 2004; Short et al., 2011; Steinberg et al., 2015). In addition, the *Fusarium* biofilm formation has been reported in contact lens wearers and patients with keratitis (Jureen et al., 2008).

Biofilms are cellular communities attached to the biotic or abiotic solid surface, which produce extracellular polymeric substances forming a gelatinous network that immobilizes and protects inside cells (Abdallah et al., 2014). Biofilm-forming microorganisms have greater resistance to antimicrobial agents and can survive after conventional disinfection procedures (Bridier et al., 2011). The cause of this resistance includes the presence of microorganisms in the biofilm innermost layers with metabolic and growth rates reduced. The extracellular polymeric matrix can act as an adsorbent, reducing the amount of antimicrobial available against the biofilm cells (Gilbert et al., 2002) or acting as an electron donor/receiver that cause the disinfectant inactivation (Bridier et al., 2011).

In medical devices, the disinfectant’s incorrect use, for example, in erroneous doses, shorter exposure time or after non-compliance with conservation guidelines, can contribute to the microorganism’s persistence that may allow the biofilm formation (Bremer, 2006).

Several disinfectants such as acetic, citric, peracetic acids, sodium hypochlorite, and sodium metabisulfite have been used in the hemodialysis microenvironment for different purposes (Sakuma et al., 2010; Oliveira et al., 2011; Bolasco et al., 2012; Kawanishi et al., 2016). National and international guidelines recommend that aqueous sodium hypochlorite solutions at 0.05%, which corresponds to 500 parts per million (ppm) with 30 minutes exposure period, are used to disinfect hemodialysis machines (API, 2010; Brazil, 2014; Rutala and Weber, 2015). In addition, 0.1% sodium hypochlorite has also been used to disinfect hydraulic pipes and treated water storage tanks monthly (Santos et al., 2007; Rutala and Weber, 2015). Disadvantages of hypochlorites such as corrosiveness to metals, inactivation by organic matter, release of chloramines when mixed with ammonia or acid and relative stability have contributed to the discontinuation of the use of hypochlorite (Rutala and Weber, 2015).

The peracetic acid in mixtures with acetic acid, hydrogen peroxide, a stabilizer, and, sometimes, sulfuric acid has increased its use frequency in the dialysis setting (Brazil, 2014; Rutala and Weber, 2015). In patient shifts, this disinfectant was used to the hemodialysis filters sterilization to allow their reuse and to the hemodialysis machines disinfection (Oliveira et al., 2011). In dialysis facilities that perform reuse, the dialyzer is specific for the patient who can reuse it up to 15 times, on average (Oliveira et al., 2011; Kreepala et al., 2017). Lacson et al. (2011) reported that factors such as exposure to the reused membrane, residual reagents, altered membrane permeability to toxins, or some combination of these alternatives would contribute to the inflammatory reaction and the common nutritional manifestations observed in patients with chronic kidney disease. Thus, the residual peroxide levels must be measured to provide safety to patients (Brazil, 2004).

To our knowledge, published studies reporting residual toxicity of acute or chronically peracetic acid are scarce demanding studies that address this important issue for public health. In this context, this study aimed to monitor the effect of disinfectants used in the dialysis process against *Aspergillus*, and *Fusarium* isolates from a hospital hemodialysis center’s water system in planktonic and biofilm form and investigates the *in vivo* toxicity of low concentrations of peracetic acid in chronic exposures.

## Materials and Methods

### Disinfectants

The disinfectants (acetic acid, citric acid, peracetic acid, sodium hypochloride, and sodium metabisulphite) used in this study were chosen to represent those used in the hemodialysis setting for disinfecting surfaces, water lines, water storage tanks, and equipment components. At the time of the tests, these agents were diluted with distilled water in order to provide the use concentration recommended by guidelines or by the manufacturer. The resulting solution was passed through microbiological filters (22 µm pores) for sterilization.

### Fungal Samples

*Fusarium* (F1 to F14) and *Aspergillus* (A1 to A23) isolates from the Laboratory of Mycology and Environmental Diagnosis, University of Franca, SP, Brazil were tested. The fungal isolates were previously recovered from the water system of a hospital Hemodialysis Center by our group (Varo et al., 2007; Pires-Gonçalves et al., 2008; Montanari et al., 2009). Fungi were grown on potato dextrose agar (PDA, Acumedia^®^, Michigan, EUA) during five-seven days at 30 °C.

### Molecular Samples Identification

#### DNA Extraction

Fungal samples were transferred to PDA and incubated at 30 °C. Conidia and cells were harvested with buffered saline (PBS - 10 mM potassium phosphate; 0.15 M NaCl, pH 7.0) for cell wall disruption in MagNA Lyser (Roche Life Science, SP, Brazil) at 5,000 × *g* for 5 min, using Magna Lyser Green Beads (Roche Life Science). DNA was extracted from this homogenized mixture using the QiaAmp DNA Mini Kit (Qiagen, Germantown, MD, USA), according to the manufacturer’s instructions.

#### PCR Reactions

PCR was performed using specific primers to amplify TEF1α (translation elongation factor— 1α) and rDNA (ribosomal DNA) genes for the identification of species of *Fusarium* and ß-tubulin and Calmodulin genes for *Aspergillus* spp. The sequences of the primers used in this study are described in **Table 1**. PCR was performed using the PCR Master Mix (Promega, Fitchburg, WI, USA). PCR reactions were incubated in a ProFlex PCR System thermocycler (Applied Biosystem, Waltham, MA, USA) under the following conditions: 2 min of initial denaturation at 95°C, 40 cycles of DNA denaturation at 95°C for 30 s, primer annealing temperature varying according to the target gene for 30 s, elongation at 72°C for 1 min and a final elongation step at 72°C for 5 min. The annealing temperatures used for TEF1α and rDNA (PCR using primers ITS5 and NL4) were 55 °C for both, and for ß-tubulin and Calmodulin, were 58 °C and 53°C, respectively. PCR products were verified by electrophoresis in a 2% agarose gel, 100 v for 30 min. PCR products were purified with ExoSAP-IT for PCR Product Clean-up (Affymetrix USB, USA) before sequencing analysis.

**Table 1:** The sequence of primers used for sequencing environmental *Fusarium* and *Aspergillus* isolates.

Species	Gene	Protein	Primer		Reference
			Name	Sequence (5´-3’)^a^	
*Fusarium* spp.	*TEF1α*	Translation elongation factor 1 alpha	HS392	TCAAAATGGGTAAGGA(A/G)GACAAGAC	O’Donnell et al., 1998; Muraosa et al., 2014
			HS393	GCCTGGGA(A/G)GTACCAGT(C/G)ATCATGTT	O’Donnell et al., 1998; Muraosa et al., 2014
			EF11	GTGGGGCATTTACCCCGCC	O’Donnell et al., 1998
			EF21	GAGTGGCGGGGTAAATGCC	O’Donnell et al., 1998
	rDNA	Ribosomal DNA	ITS4	TCCTCCGCTTATTGATATGC	Scheel et al., 2013
			ITS5	GGAAGTAAAAGTCGTAACAAGG	O’Donnell et al., 2009
			NL1	GCATATCAATAAGCGGAGGAAAAG	Scheel et al., 2013
			NL4	GGTCCGTGTTTCAAGACGG	O’Donnell et al., 2009
*Aspergillus* spp.	*ß-tubulin*	ß-tubulin	2A	GGTAACCAAATCGGTGCTGCTTTC	Glass and Donaldson, 1995
			2B	ACCCTCAGTGTAGTGACCCTTGGC	Glass and Donaldson, 1995
	*Calmodulin*	Calmodulin	CMD5	CCGAGTACAAGGAGGCCTTC	Hong et al., 2005
			CMD6	CCGATAGAGGTCATAACGTGG	Hong et al., 2005

a Y: C or T; W: A or T; R: A or G; K: G or T; S: G or C; D: A, G or T; B: G, T or C; H: A, C or T.

#### DNA Sequencing for Identification

A partial portion of TEF1α was sequenced with the BigDye Terminator reagent kit (Applied Biosystems) in a SeqStudio Genetic Analyzer (Applied Biosystems) using HS392, HS393, EF11, and EF21 primers (O’Donnell et al., 1998; Scheel et al., 2013; Muraosa et al., 2014; Moretti et al., 2018). The portion rDNA was sequenced using the primers ITS4, ITS5, NL1, and NL4 (**Table 1**). DNA sequences were edited and assembled by Sequencher version 5.2.4 (Gene Codes, USA). For identification, a homology search for the sequences of TEF1α and rDNA genes was done using the BLAST tool of the NCBI database (GenBank), the database FUSARIUM-ID (https://isolate.fusariumdb.orgl/), and the Fusarium MLST (http://www.cbs.knaw.nl/fusarium). ß-tubulin and Calmodulin genes were sequenced and used for the pairwise alignment in the NCBI database (GenBank) for the identification of species of *Aspergillus* (Glass and Donaldson, 1995; Hong et al., 2005). The sequence of primers used are described in **Table 1**.

### Susceptibility Test of Planktonic Cells to Disinfectants

All fungal samples [*Aspergillus* spp. (n=19) and *Fusarium* spp. (n=13)] were grown on PDA for 4-5 days at 30 °C for conidia production. After that time, 5 ml of sterile PBS containing 0.025% (v/v) of Tween 20 (Santos et al., 2007; Pierce et al., 2008) was added and manually stirred. The resulting conidial suspension was filtered through filter paper to remove debris. The broth microdilution method was performed according to the protocol M38-A2 from the Clinical and Laboratory Standard Institute (CLSI, 2008), with modifications adapted to disinfectants. An aliquot (100 µl) of the fungal inoculum with turbidity equivalent to the standard 0.5 McFarland was diluted 1:50 in RPMI-1640 medium (Memorial Roswell Park Institute - Sigma Chemical Co., Steinheim, Germany), buffered at pH 7.0 with MOPS (3-morpholin-4-yl-propane-1-sulfonic acid, Sigma), added with 0.2% glucose and inoculated into 96-well microtiter plates. Follow, 100 µl of the diluted disinfectant were added to the wells at pre-determined concentration according to the guidelines or the manufacturer. The microplates were incubated at 30°C for a specific time for each disinfectant or concentration tested (Mattei et al., 2013; Groote et al., 2014). A fungal inoculum control well without the disinfectant and a well with only the disinfectant and culture medium were prepared for each disinfectant/concentration tested. After the incubation time, the total content of each well was transferred to 10 ml of buffered saline (PBS), allowing an initial dilution of 1:50. Decimal dilutions were performed in PBS and the antifungal activity of each disinfectant was assessed by plating 100 μl in SDA, incubated at 30° C for up to seven days. The number of Colony Forming Units (CFU) per milliliter (ml) was calculated from the number of colonies formed multiplied by the dilution factor (Wilson et al., 2017). A chemical disinfecting agent is expected to be capable of a 3 Log_10_ reduction of the infectious bacteria from the initial inoculum (Rutala, 1995). As values for fungi are not established, we adopt the same criterion. Each fungal isolated was tested individually and the arithmetic mean of the CFU/ml obtained was used for comparative analysis. The experiments were carried out in triplicate and on three different occasions.

### Biofilm Assays

#### Biofilm Formation

Biofilms were formed in 24 well-microtiter plates which contained 13mm diameter circular glass coverslips. A 1 ml aliquot of the standardized inoculum at 10^5^ cells/ml in RPMI medium was added to each well and incubated for 72 hours at 37°C (Pierce et al., 2008; Seidler et al., 2008). The biofilms were then washed with PBS (3x) and fixed with 0.1M potassium phosphate buffer solution added with 2.5% glutaraldehyde and 4% paraformaldehyde overnight. This was followed by washing the biofilms with phosphate buffer and post-fixation treatment with osmium tetroxide 1% for 16h. After new washes, the biofilms were dehydrated with absolute ethyl alcohol from a gradient of 30% to 100% for 30 minutes each. Finally, the biofilms were dried in a critical point chamber (MS 850, Electron Microscopy Sciences), mounted on aluminum, metallized with gold (Denton Vacuum Desk II coater) and observed under a scanning electron microscope (JSM 5410: JEOL, Tokyo, Japan).

#### Susceptibility Test of Sessile Cells (Biofilms) to Disinfectants

At the time of testing, all conidial suspensions from *Aspergillus* and *Fusarium* strains were adjusted to a cell concentration of 10^5^ cells/ml (Pierce et al., 2008), in standardized culture medium (RPMI 1640). Then, 200 µl of each suspension was dispensed in 96-well microtiter plates (TPP, BIOGEN, Europe) and incubated at 37°C for 72 h (Pierce et al., 2008; Seidler et al., 2008). Wells with only culture medium were included in the assay as a control of reaction sterility as well as wells with inoculum in RPMI as growth controls (100% cell viability). Then, the supernatant from the wells containing the biofilms was carefully aspirated and washed with PBS three times to remove non-adherent cells. Dilutions of disinfectant were freshly prepared and 100 µl were added to the preformed biofilms, incubating again for a specific time for each disinfectant/concentration at 30 °C (lknur et al., 2012). After the incubation time, the biofilms were subjected to cell viability tests. Each fungal sample was tested individually in triplicate on three different occasions.

#### Evaluation of Biofilms Cell Viability

After supernatant aspiration and washing the biofilms, they were subjected to the cell viability quantification by the reducing the tetrazolium salt 3’- [1- (phenylaminocarbonyl] - 3,4-tetrazolium] -bis (4-methoxy-6 -nitro) hydrated benzene sulfonic acid (XTT) methodology (Pierce et al., 2008). Briefly, XTT (Sigma, St.Louis, Mo., USA) was prepared at a concentration of 0.5 g/l in PBS and filtered (0.22 micrometers in size). At the time of the test, menadione (Sigma) was added to the 1 µM final concentration. The plates were incubated in the dark for 4 h at 37°C and the colorimetric change measured in a plate reader at 492 nm. Wells containing culture medium/biofilms/XTT/menadione (positive control) and wells containing culture medium/XTT/menadione (negative control) were included. No standardization for assessing the disinfectants sensitivity against fungal biofilms was found. Thus, the criterion of 80% metabolic activity inhibition when compared to control (without disinfectant) was adopted, as previously published by Van Dijck et al. (2018). Each fungal sample was tested individually and the arithmetic mean of the optical densities obtained was used for comparative analysis. The experiments were carried out in triplicate on three different occasions.

### Chronic Toxicity of Peracetic Acid in Mice

Among all disinfectants used in the hemodialysis environment, the peracetic acid-based disinfectants has been widely used both for the disinfection of machines between patient shifts and for the conditioning of dialyzers (reuse) between dialysis sessions (Oliveira et al., 2011; Szewczyk et al., 2013; Rutala and Weber, 2015). Factors such as: decomposition into non-toxic and non-mutagenic products; efficiency even in the presence of organic residues; effectiveness in low doses and against a wide spectrum of antimicrobial agents have contributed to the choice of peracetic acid to replace sodium hypochlorite (Rutala and Weber, 2015). In addition, the detection of its bioproduct - acetic acid, at the level of 1 ppm must be measured in the fluids of the hemodialysis machine prior to the patient’s connection to it (Oliveira et al., 2011). Thus, we chose peracetic acid 1 ppm to perform the chronic exposure tests in mice.

#### Animals Maintenance

The experiments were conducted according to ethical recommendations and after approval by the Ethics Committee on the Use of Animals in research at the University of Franca, under the protocol number (8017291118 (ID 000718). Healthy, adult female mice of the BALB/c lineage, weighing between 20-24 grams (8-9 weeks of age) were used. The animals were accommodated in cages and submitted to environment with controlled temperature (22+/-2°C), humidity (40-50%), a dark/light cycle with artificial light for a 12-hour period a day, ad libidum water supply and a diet composed of rodent food. The experiments were carried out between 10:00 and 12:00 hours to avoid circadian variation. The animals were randomly divided into three groups: a) Group 1 (Control group - CT): consisting of five animals, which were not subjected to any intervention; b) Group 2 (Saline group- SS): consisting of 10 animals that were inoculated, each, in the peritoneal cavity between the abdominal organs, 20 µl of physiological saline solution (NaCl 0.9%, w/v); c) Group 3 (Disinfectant group - DSF): consisting of 10 animals, which were injected, intraperitoneally, 20 µl of 1 ppm peracetic acid solution. All treatments were performed three times a week (same periodicity as the dialysis treatment), in groups SS and DSF during 60 weeks, using 25x5mm needles. The animals were monitored daily, observing the parameters of behavior, locomotion, breathing, skin/fur changes, eyes, tremors, salivation, lethargy and survival. One week after the last treatment, the animals were weighed, anesthetized with sodium thiopental 0,84 g/kg, intraperitoneally, right lower lateral quadrant, and blood samples were collected to determine hematological and biochemical parameters.

#### Histological Analysis

Lung, liver, spleen, heart and brain were removed from five animals of each group for histological evaluation (n=15). After necropsy, the organs were weighed and their characteristics were noted. Samples (10 mm long x 1.5 mm thick) were collected from these organs and fixed in 10% buffered formaldehyde for histopathological analysis. The tissue cuts were dehydrated by means of an increasing degree of ethanol (70-100%), diaphanized in xylol, impregnated and included in paraffin. The blocks were cut in a microtome adjusted to 3 μm. The cuts obtained were placed on slides smeared with albumin and drying at 37 °C for 24 hours. The sections were subjected to Masson’s trichrome staining technique and analyzed under a light microscope, under fixed focus and field clarity, with a final magnification of 100X and 400X. Descriptive analysis of the results was performed. Aiming to reduce the margin of error in histological analysis, the samples were evaluated and classified separately, blindly, by three pathologists (AWO., SAJr., LCZ), with a later discussion of the cases with disagreement. The agreement between at least two pathologists defined the sample final classification (Lee et al., 2018).

#### Biochemical and Hematological Parameters

To determine the biochemical profile, 200 µl of blood was collected from each animal in 1.5 ml eppendorfs, previously identified and centrifuged at 4,750 g for 10 minutes at room temperature to obtain the serum. Samples that showed hemolysis were discarded. Serum levels of albumin, alanine amino transferase (ALT), and aspartate amino transferase (AST) were determined. For biochemical tests, commercial reagents were used (Labtest^®^, Labtest Diagnóstica S. A., Lagoa Santa, MG, Brazil), performed on a spectrophotometer (Mindray BS-200, Shenzhen, China). The reference values considered were: ALT: 94.0 – 188.0 U/l; AST: 89.0 – 172.0 U/l (Araújo, 2012).

From the total volume of blood collected from each animal, approximately 50µl were removed and packed in 1.5ml eppendorfs, previously identified, containing 5 µl of EDTA (Ethylene diamine tetraacetic) as an anticoagulant. The determinations were performed in a semi-automatic hematological cell conter (ABX Micros ABC Vet), suitable for measuring veterinary hematological parameters. To check the equipment calibration, ABX Minotrol 16 high (H), normal (N) and low (L) control blood was used prior to the start of each experiment. Red blood cells (RBC), hematocrit (HCT) and hemoglobin (Hb), white blood cells (WBC) and platelets (PLT) were also quantified. The reference values considered were: RBC: 7.2 - 11.2x10^6^/mm^3^; HCT: 33.1 - 52.0%; Hb: 10.3 - 16.6 g/dl; WBC: 1.0 - 5.5x10^3^/mm^3^; PLT: 439.0 - 957.0x10^3^/mm^3^ (Araújo, 2012).

### Statistical Analysis

Statistical analyses were performed using the software GraphPad Prism 7.0 (GraphPad Software Incp., San Diego, CA). The data were analyzed for normality (Kolmogorov–Smirnov), and the one-way ANOVA test will be used followed by the Tukey test as a post-test. To compare data between animals groups, the Student t test (parametric) was used. For values of p <0.05, the data will be considered statistically significant.

## Results

### Fungal Identification

**Figure 1:**
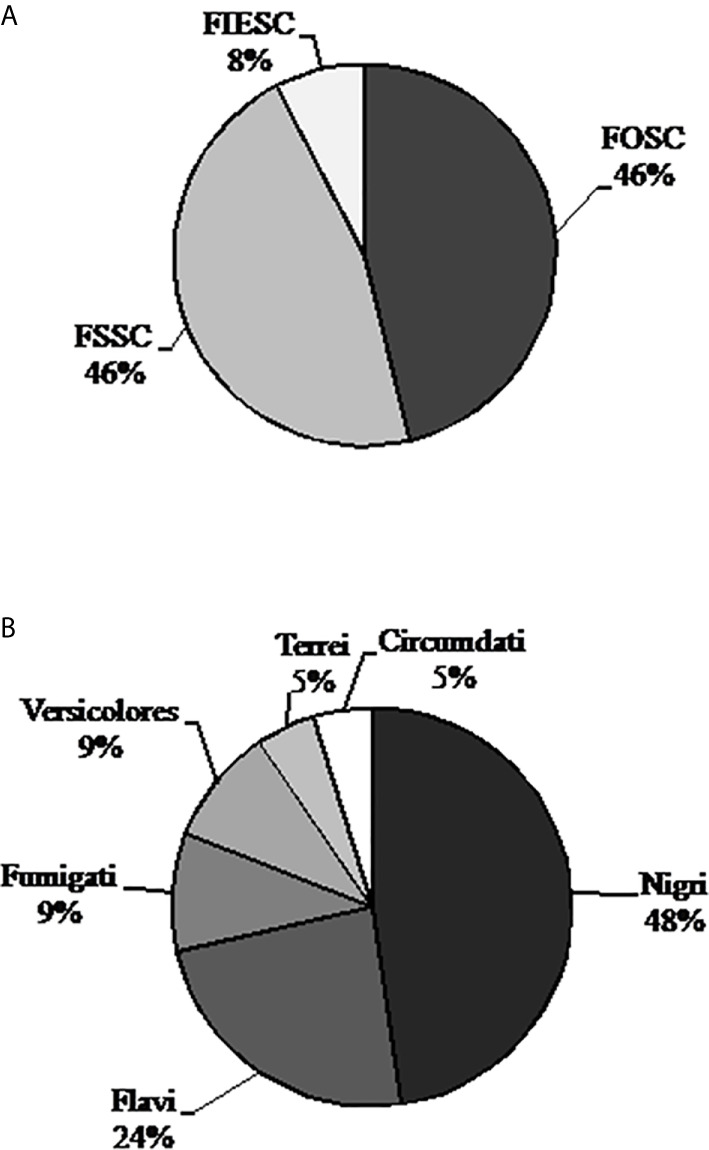
Distribution of the *Fusarium* **(A)** and *Aspergillus* **(B)** strains among complexes or sections. Legend: FOSC - *Fusarium oxysporum* species complex, FSSC - *Fusarium solani* species complex, FIESC - *Fusarium incarnatum-equiseti* species complex.

DNA sequencing of 13 strains of *Fusarium* was identified as FOSC - *Fusarium oxysporum* species complex (46,1%), FSSC - *Fusarium solani* species complex (46,1%) and FIESC - *Fusarium incarnatum-equiseti* species complex (7,7%). Regarding the 21 strains of *Aspergillus* (**Figure 1**), 10 were identified as *Nigri* section (47,6%), five as *Flavi* (23,8%), two as *Fumigati* (9,5%), two as *Versicolores* (9,5%), one as *Terrei* (4,7%), and one as *Circumdati* (4,7%). Two strains were identified as *Penicillum* spp., probably due to contamination (data not shown). Molecular identification of the strains at the species level within each complex or section was possible after sequencing using more than one gene as described in **Table 2**. To identify *Fusarium*, TEF1α gene and rDNA region were used, but more genomic loci as RPB1 (largest subunit of RNA polymerase) and RPB2 (second largest subunit of RNA polymerase) can be used for phylogenetic analysis and obtaining a more precise classification. Although specific databases were used to identify *Fusarium*, the species was shown only in the NCBI gene bank. For *Aspergillus* spp., the species were identified after Calmodulin gene sequencing, searching only in the NCBI database.

**Table 2:** Molecular identification of species of *Fusarium* and *Aspergillus*.

ID	Molecular Identification	Complex/Section
F1	*Fusarium oxysporum*	FOSC
F3	*Fusarium solani*	FSSC
F4	*Fusarium oxysporum*	FOSC
F5	*Fusarium oxysporum*	FOSC
F6	*Fusarium solani*	FSSC
F7	*Fusarium solani*	FSSC
F8	*Fusarium oxysporum*	FOSC
F9	*Fusarium solani*	FSSC
F10	*Fusarium oxysporum*	FOSC
F11	*Fusarium equiseti*	FIESC
F12	*Fusarium oxysporum*	FOSC
F13	*Fusarium solani*	FSSC
F14	*Fusarium solani*	FSSC
A1	*Aspergillus niger*	Nigri
A3	*Aspergillus fumigatus*	Fumigati
A4	*Aspergillus terreus*	Terrei
A5	*Aspergillus niger*	Nigri
A6	*Aspergillus niger*	Nigri
A7	*Aspergillus tamarii*	Flavi
A8	*Aspergillus tamarii*	Flavi
A9	*Aspergillus sclerotiorum*	Circumdati
A10	*Aspergillus niger*	Nigri
A11	*Aspergillus niger*	Nigri
A12	*Aspergillus niger*	Nigri
A13	*Aspergillus niger*	Nigri
A14	*Aspergillus tamarii*	Flavi
A16	*Aspergillus sydowii*	Versicolores
A17	*Aspergillus niger*	Nigri
A18	*Aspergillus niger*	Nigri
A19	*Aspergillus tamarii*	Flavi
A20	*Aspergillus sydowii*	Versicolores
A21	*Aspergillus fumigatus*	Fumigati
A22	*Aspergillus niger*	Nigri
A23	*Aspergillus tamarii*	Flavi

FOSC, Fusarium oxysporum species complex; FSSC, Fusarium solani species complex; FIESC, Fusarium incarnatum-equiseti species complex.

### Disinfectant Activity on Planktonic Cells

The results of the tests performed with *Fusarium* fungal cells in free or suspended form, called planktonic cells, are shown in **Table 3**. Both 1% acetic acid and 2.5% sodium hypochlorite exerted a fungicidal action, since they inhibited fungal growth at 4.2 Log_10_ cells/ml and 4.24 Log_10_ cells/ml, respectively. The other disinfectants inhibited fungal growth, on average, 1.22 Log_10_ cells/ml. In the case of *Aspergillus* (**Table 4**), 2.5% sodium hypochlorite enabled the inactivation of 4.38 Log_10_ cells/ml, followed by 0.1% peracetic acid that reduced 1.76 Log_10_ cells/ml. The other disinfectants tested inhibited fungal growth by approximately 1.09 Log_10_ cells/ml. Thus, 2.5% sodium hypochlorite was the only fungicidal agent for *Aspergillus* in planktonic growth.

**Table 3:** Disinfectants activities against *Fusarium* planctonic cells.

Disinfectant	Origin	Final concentration tested	Exposure time	Control (CFU/ml ± SD)	Control (Log_10_)	Treated (CFU/ml ± SD)	Treated (Log_10_)
Glacial acetic acid	Synth, SP, Brazil	1% (v/v)	30 min	1.59x10^4^ ± 2.5x10^2^	4.2	**0**	**0**
Citric acid	Bell Citric 50^®^, Presidente Prudente, SP, Brazil	1% (v/v)	120 min	1.56x10^4^ ± 2.1x10^2^	4.19	6.15x10^2^ ± 1.42x10	2.78
		21% (v/v)	40 min	1.96x10^4^ ± 1.94x10^2^	4.29	7.18x10^2^ ± 1.34x10	2.85
		50% (v/v)	15 min	1.80x10^4^ ± 2.3x10^2^	4.25	3.96x10^2^ ± 1.10x10	2.59
Peracetic acid	Puristeril 340^®^, Fresenius Medical Care, Jaguariuna, SP, Brazil	0.1% (v/v)	30 min	1.88x10^4^ ± 2.2x10^2^	4.27	1.10x10^3^ ± 2.10x10^2^	3.03
		0.2% (v/v)	10 min	1.96x10^4^ ± 1.8x10^2^	4.29	2.08x10^3^ ± 3.04x10^2^	3.31
Sodium hypochlorite	12%, Solynt, Guarulhos, SP, Brazil	0.05% (v/v)	30 min	1.76x10^4^ ± 2.4x10^2^	4.24	1.18x10^3^ ± 4.20x10^2^	3.07
		0.1% (v/v)	10 min	1.82x10^4^ ± 2.1x10^2^	4.26	1.18x10^3^ ± 3.84x10^2^	3.07
		2.5% (v/v)	30 min	1.76x10^4^ ± 2.3x10^2^	4.24	**0**	**0**
Sodium metabisulphite	Synth, SP, Brazil	0.1% (w/v)	120 min	1.64x10^4^ ± 1.9x10^2^	4.21	2.92x10^3^ ± 4.14x10^2^	3.46

CFU/ml, Colony Forming Unit per milliliter. Bold numbers denote disinfectant activity.

**Table 4:** Disinfectants activities against *Aspergillus* plactonic cells.

Disinfectant	Origin	Final concentration tested	Exposure time	Control (CFU/ml ± SD)	Control (Log_10_)	Treated (CFU/ml ± SD)	Treated (Log_10_)
Glacial acetic acid	Synth, SP, Brazil	1% (v/v)	30 min	2.5x10^4^ ± 2.1x10^3^	4.39	2.44x10^3^ ± 2.46x10^2^	3.43
Citric acid	Bell Citric 50^®^, Presidente Prudente, SP, Brazil	1% (v/v)	120 min	2.4x10^4^ ± 2.6x10^3^	4.38	1.89x10^3^ ± 2.08x10^2^	3.27
		21% (v/v)	40 min	2.6x10^4^ ± 2.5x10^3^	4.41	1.70x10^3^ ± 3.21x10^2^	3.22
		50% (v/v)	15 min	2.5x10^4^ ± 1.7x10^3^	4.39	1.08x10^3^ ± 1.53x10^2^	3.03
Peracetic acid	Puristeril 340^®^, Fresenius Medical Care, Jaguariuna, SP, Brazil	0.1% (v/v)	30 min	2.4x10^4^ ± 2.2x10^3^	4.38	**4.17x10^2^ ± 1.81x10^2^**	2.62
		0.2% (v/v)	10 min	2.5x10^4^ ± 1.7x10^3^	4.39	1.48x10^3^ ± 2.24x10^2^	3.17
Sodium hypochlorite	12%, Solynt, Guarulhos, SP, Brazil	0.05% (v/v)	30 min	2.5x10^4^ ± 1.6x10^3^	4.39	4.36x10^3^ ± 3.51x10^2^	3.63
		0.1% (v/v)	10 min	2.5x10^4^ ± 1.6x10^3^	4.39	3.28x10^3^ ± 3.70x10^2^	3.51
		2.5% (v/v)	30 min	2.4x10^4^ ± 1.5x10^3^	4.38	**0**	**0**
Sodium metabisulphite	Synth, SP, Brazil	0.1% (w/v)	120 min	2.4x10^4^ ± 1.5x10^3^	4.38	1.23x10^3^ ± 2.44x10^2^	3.09

CFU/ml, Colony Forming Unit per milliliter; Bold numbers denote disinfectant activity.

### Demonstration of Biofilms Using Scanning Electron Microscopy

**Figure 2:**
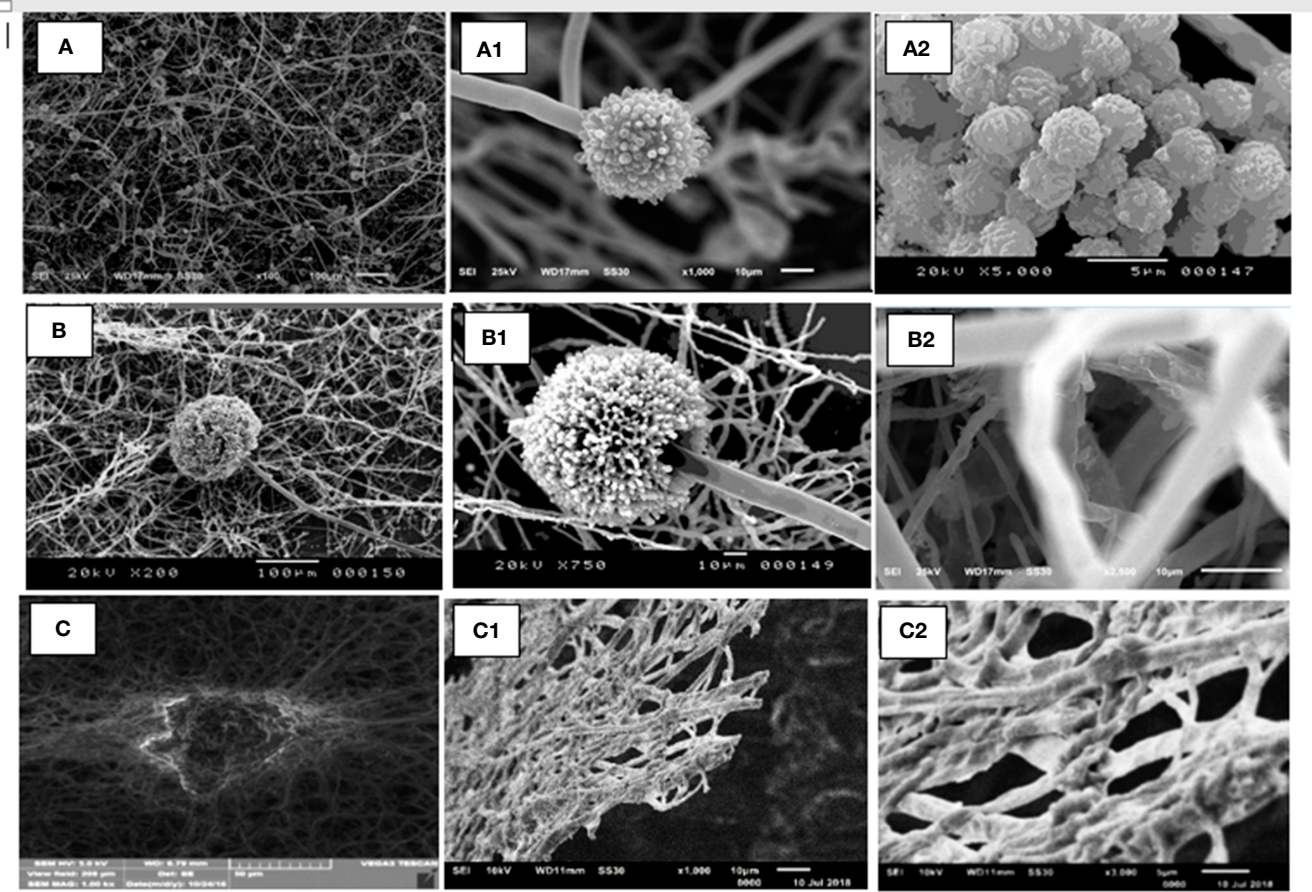
Representative micrographs of the *Aspergillus* (**A** – A.niger; **B** – *A. terreus*) and *Fusarium oxysporum* **(C)** biofilms.

Representative electron micrographs showed robust *Aspergillus* biofilms (**Figures 2A** - A*. niger*, 2B - A*. terreus*), with intertwined hyphae, forming overlapping layers (2A; 2B). Conidial heads were observed in all *Aspergillus* biofilms (2A1; 2B1). Dense matrix covering the conidia (2A2) or the hyphae (2B2) were also observed. *F. oxysporum* biofilms (C) showed the extensive formation of interlaced hyphae (C1) covered by matrix (C2).

### Disinfectant Activity on Sessile (Biofilm) Cells

Quantification of biofilms was performed by colorimetric XTT reduction assay and viability was expressed in terms of percentage metabolic activity. Against *Fusarium* spp. biofilms, the disinfectants citric acid (50%, 15min), peracetic acid (0.1%, 30 min) and sodium hypochlorite (2.5%, 30 min) were effective (**Table 5**). Besides, acetic acid (1%, 30 min), citric acid (50%, 15 min), peracetic acid (0.2%, 10 min), and sodium hypochlorite (2.5%, 30 min) showed effectiveness against *Aspergillus* spp. biofilms (**Table 5**).

**Table 5:** Disinfectants activities against *Aspergillus* and *Fusarium* biofilm cells.

Disinfectant	Origin	Final concentration tested	Exposure time	*Fusarium*		*Aspergillus*	
				Control (OD 492nm)	Treated (OD 492nm)	Control (OD 492nm)	Treated (OD 492nm)
Glacial acetic acid	Synth, SP, Brazil	1% (v/v)	30 min	1.45 ± 0.08	0.40 ± 0.06	1.82 ± 0.07	**0.22 ± 0.01**
Citric acid	Bell Citric 50^®^, Presidente Prudente, SP, Brazil	1% (v/v)	120 min	1.42 ± 0.11	0.70 ± 0.06	1.80 ± 0.03	0.87 ± 0.01
		21% (v/v)	40 min	1.44 ± 0.08	0.53 ± 0.13	1.84 ± 0.16	0.72 ± 0.10
		50% (v/v)	15 min	1.42 ± 0.09	**0.10 ± 0.04**	1.82 ± 0.08	**0.31 ± 0.05**
Peracetic acid	Puristeril 340^®^, Fresenius Medical Care, Jaguariuna, SP, Brazil	0.1% (v/v)	30 min	1.41 ± 0.13	**0.37 ± 0.11**	1.82 ± 0.21	0.43 ± 0.11
		0.2% (v/v)	10 min	1.41 ± 0.09	**0.16 ± 0.04**	1.81 ± 0.17	**0.35 ± 0.11**
Sodium hypochlorite	12%, Solynt, Guarulhos, SP, Brazil	0.05% (v/v)	30 min	1.46 ± 0.14	0.77 ± 0.09	1.84 ± 0.26	0.97 ± 0.10
		0.1% (v/v)	10 min	1.40 ± 0.20	0.73 ± 0.02	1.81 ± 0.23	0.77 ± 0.15
		2.5% (v/v)	30 min	1.45 ± 0.14	**0.03 ± 0.05**	1.81 ± 0.19	**0.06 ± 0.04**
Sodium metabisulphite	Synth, SP, Brazil	0.1% (w/v)	120 min	1.45 ± 0.24	0.69 ± 0.18	1.82 ± 0.13	0.50 ± 0.10

Bold numbers denote 80% reduction or more when compared to control.

### Histological Analysis of Murine Organs

The BALB/c mice were weighed before and after the experiments. In the experimental period (60 weeks) there was no significant difference in the animals’ weight gain. The weight variation obtained was 6.8 ± 2.1g; 7.4 ± 1.4g and 6.2 ± 0.5g for the CT, SS and DSF groups, respectively. At the necropsy, there were no changes in the area of body coverage with fur, spots, and erythema. Mucous membranes had a normal appearance and the lymph nodes, which did not show changes in volume or color, and ocular region with no changes and no secretion in the nasal area. There were no statistically significant changes in the weight of lung, brain, kidney, heart, and spleen in the animal groups (p> 0.05). However, concerning the liver, the group treated with paracetic acid had organ-to-body weight ratios of 1.068, significantly different from group CT that presents a 1.206 ratio (p=0.0002). The comparative histopathological data between the organs are described in **Table 6**, with slight changes in liver, lung, spleen, and pancreas architecture.

**Table 6:** Comparative summary of histopathological analysis between the different organs of the animals groups studied (n = 15).

Organ	Architecture			Inflammation			Necrosis			Hemorrhage			Congestion			Hyperemia		
	CT	SS	DSF	CT	SS	DSF	CT	SS	DSF	CT	SS	DSF	CT	SS	DSF	CT	SS	DSF
**Liver**	Normal	Normal	Discrete degeneration hydropic centrolobular	Absent	Absent	Absent	Absent	Absent	Absent	Absent	Absent	Absent	Absent	Absent	Discreet and multifocal	Absent	Absent	Absent
**Brain**	Normal	Normal	Normal	Absent	Absent	Absent	Absent	Absent	Absent	Absent	Absent	Absent	Absent	Absent	Absent	Absent	Absent	Absent
**Heart**	Normal	Normal	Normal	Absent	Absent	Absent	Absent	Absent	Absent	Absent	Absent	Absent	Absent	Absent	Absent	Absent	Absent	Absent
**Lung**	Normal	Normal	Emphysema areas in fragment edges	Absent	Absent	Absent	Absent	Absent	Absent	Absent	Absent	Absent	Absent	Discreet and diffuse	Discreet and multifocal	Absent	Absent	Discreet
**Kidney**	Normal	Normal	Normal	Absent	Absent	Absent	Absent	Absent	Discreet tubular necrosis	Absent	Absent	Absent	Absent	Absent	Absent	Absent	Absent	Absent
**Spleen**	Normal	Normal	Lymphoid hyperplasia follicular, with expansion the germinal center and marginal zone, for times with coalescence	Absent	Absent	Absent	Absent	Absent	Absent	Absent	Absent	Absent	Absent	Discreet and diffuse	Discreet and diffuse	Absent	Absent	Discreet
**Pancreas**	Normal	Normal	Follicular hyperplasia	Absent	Absent	Absent	Absent	Absent	Absent	Absent	Absent	Absent	Absent	Absent	Absent	Absent	Absent	Absent

Groups: CT, Control; SS, Saline Solution; DSF, Disinfectant.

### Determination of Biochemical and Hematological Parameters

**Figure 3:**
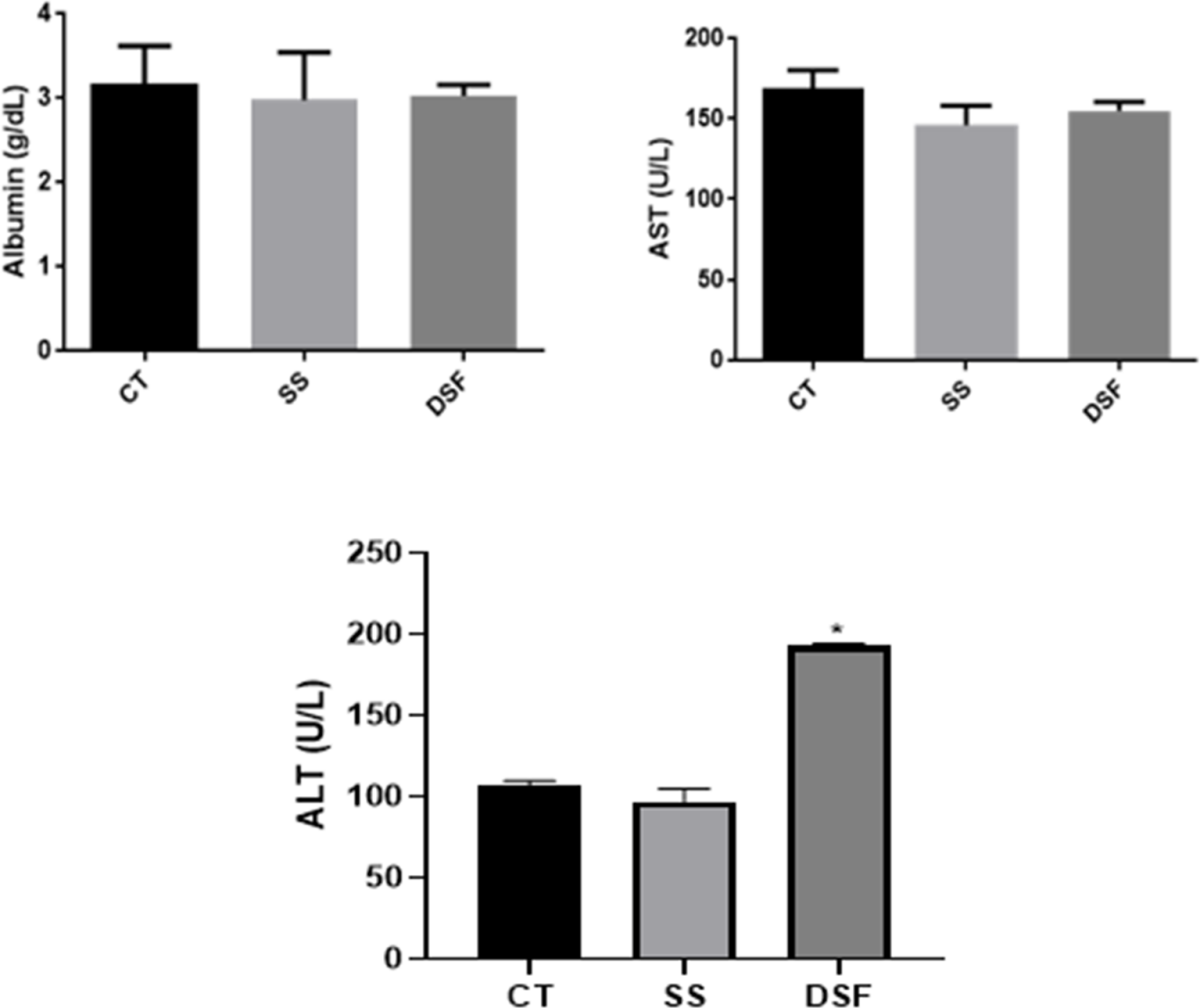
Biochemical values obtained for the groups of mice studied (CT = control group; SS = treated with sterile saline and DSF = group treated with 1 ppm peracetic acid). The results are expressed as mean and standard deviation. *p < 0.05.

**Figure 4:**
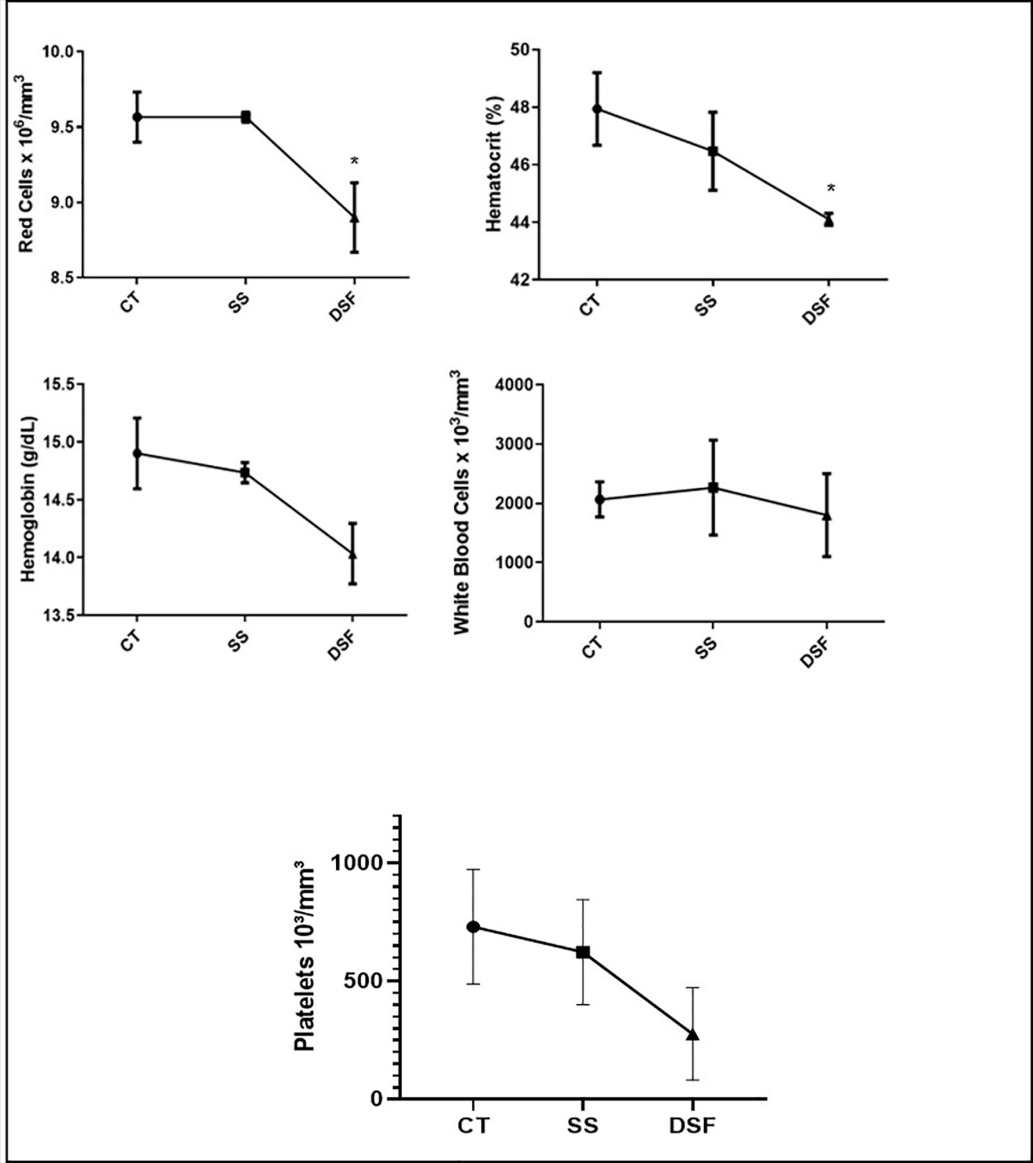
Hematological values obtained for the groups of mice studied (CT = control group; SS = treated with sterile saline and DSF = group treated with 1 ppm peracetic acid). The results are expressed as mean and standard deviation. *p < 0.05.

The biochemical analysis of BALB/c female mice are presented in **Figure 3**. ALT liver enzyme had higher-level values than the other groups (p ≤ 0.05), denoting liver injury. The hematological values determined for the animal groups are shown in **Figure 4**. The comparison between the CT and SS group or DSF was performed using the Student t-test. There was a significant difference between the RBC (p<0.05) and HCT (p <0.05) values when comparing the CT group and DSF group (**Figure 4**). When the DSF group was compared to the CT group, no significant difference was found for hemoglobin and platelets, although lower values were observed for the first group (**Figure 4**).

## Discussion

The *Fusarium* and *Aspergillus* strains identified in this study belong to relevant species associated with the pathogenicity and frequency in human infections. *F. solani* (FSSC) and the *F. oxysporum* species complex (FOSC) are considered the most clinically relevant pathogens, associated with high levels of resistance to systemic azoles, echinocandins, and polyenes (Sav et al., 2018). Concerning the *Aspergillus* strains, the species as *A. flavus, A. fumigatus, A niger* and *A. terreus* have a higher incidence in human infections (Paulussen et al., 2017). Various factors, including the production of asexual conidia (which rapidly propagate through the air and are highly tolerant to stress), sexual ascospores (environmentally persistent), mycotoxins, the ability to germinate at the lowest water activity, to generate cell-available energy, to produce a large number of proteases and enzymes, to penetrate host defenses and colonize/damage the host contribute to make *Aspergillus* the most potent opportunistic fungal pathogens of mammalian hosts (Kamei and Watanabe, 2005; Kwon-Chung and Sugui, 2013; Lackner and Lass-Flörl, 2013; Paulussen et al., 2017). Overall, infections by *Aspergillus* or *Fusarim* present considerable challenges due to the complexity of pathogenic mechanisms involving environmental and host-related factors.

Furthermore, the microbial contamination of the hemodialysis system can result in biofilm formation, which is recognized as an essential virulence factor for *Aspergillus* and *Fusarim* species (Peiqian et al., 2014; Beauvais and Latgé, 2015; Sav et al., 2018; Bamford et al., 2020). These biofilms may contribute to drains blockage, reduced flow of fluids, and the sensor’s accuracy. In addition, inadequate decontamination and washing of surfaces exposed to bicarbonate as a storage tank, delivery loop, and jars can quickly lead to biofilm formation (Desai, 2015).

The use of disinfectants in appropriate concentrations and exposure times is essential to reduce the spread of infections. Sodium hypochlorite at 0.05% (=500 ppm) and 0.1% concentrations, with 30 and 10 minutes exposure, respectively, has been recommended by regulatory agencies (Brazil, 2004; Brazil, 2014). Our results showed that sodium hypochlorite concentrations were not effective against planktonic or sessile cells of the tested fungi. It should be noted that the current standards are designed for bacteria that are comparatively more sensitive to disinfection processes than fungi (Hageskal et al., 2012). In addition, previous research has shown that fungal biofilms formed by organisms recovered from a chlorinated drinking water system, including *Fusarium* spp., did not have their development affected by 0.4–0.5 mg/l chlorine concentrations (Fish and Boxall, 2018a; Fish et al., 2018b). The same authors also used genomic tools (quantitative PCR -qPCR and sequencing) and did not identify changes in the biofilm genes exposed to these chlorine concentrations. Fungal tolerance to chlorine disinfection was attributed to its complex cell morphology and spore production (Fish et al., 2018b). Therefore, to control the fungal development and persistence, especially in biofilms, the disinfection protocols and the intervention frequency need to be reviewed.

Here, we show that 2.5% sodium hypochlorite was effective against both growth forms of *Aspergillus* spp. and *Fusarium* spp. This concentration is recommended for disinfecting surfaces (Brazil, 2004; Brazil, 2014). A study conducted by Hotchkiss et al. (2007) developed a probabilistic model to estimate the transmissibility of the pathogen in the dialysis environment. The same authors reported that simple environmental decontamination (primarily from the chair where the patient sits to receive hemodialysis treatment) coupled with temporal segregation (schedule of patients with suspected infections with high transmission power) for dialysis shifts at the end of the day would significantly mitigate the pathogens spread. Low-cost and straightforward measures could contribute to substantial decreases in infection rates in chronic renal patients, which reached 2.5 million people in 2015 and is expected to reach 5.4 million by 2030 (Liyanage et al., 2015).

In addition, hemodialysis machines represent one of the most used devices in modern medicine, since hemodialysis is the therapeutic form globally applied to more than 90% of patients undergoing renal replacement therapy (Som et al., 2017; Masakane et al., 2018; Thomé et al., 2019; Elshahat et al., 2020). 1% acetic acid is used to wash these machines twice a week to prevent the formation and deposition of calcium carbonate inside the equipment, preventing the adhesion and the biofilm formation (Kawanishi et al., 2016). Although it is an efficient procedure, few hemodialysis facilities use it. Our study points out that 1% acetic acid inhibits the *Fusarium* spp. planktonic growth and reduces 80% of *Aspergillus* spp. biofilms. The microbicidal property of 1% acetic acid was described in 1916, when it was used to treat war-wounds caused by *Pseudomonas aeruginosa* (Bjarnsholt et al., 2015). Currently, in a medical setting, its use has been restricted to topical use (2.5%) in dermatology (Halstead et al., 2015) and in urology (2-5%) as an urethral catheter irrigation solution (Doles et al., 2015). Anti-biofilm activity of acetic acid at 0.5% or 1% concentrations has been described to inactivate *P. aeruginosa* and *Staphylococcus aureus* after 24h of exposure (Bjarnsholt et al., 2015), and against *Candida orthopsilosis* after 1-minute exposure (Pires et al., 2013).

Citric acid at 1%, 21%, and 50% concentrations with exposure for 120, 40, and 15 minutes, respectively, has also been used in hemodialysis to dissolve carbonates. Its descaling action is superior to that of acetic acid. The combination of thermal disinfection with citric acid solution increases the antibacterial effect and removes proteins (Sakuma et al., 2010). Against planktonic fungal cells, little impact of the cited disinfectant was observed, since it was reduced by approximately 2 Log_10_ cells/ml and 1 Log_10_ cells/ml in the *Fusarium* spp. and *Aspergillus* spp. counts, respectively. Citric acid at a concentration of 50% showed an anti-biofilm effect for the tested fungi. There are reports that citrate has advantages over heparin for hemodialysis central venous catheters since it has a lower cost and more significant antibacterial activity. Such activity seems to be related to calcium and magnesium ions’ chelation, leading to the degradation of bacterial cell membranes, reducing bacterial cell integrity (Weijmer et al., 2002). The bactericidal and sporicidal activity has been reported for citrate concentrations at 4% and 23%, respectively (Ash et al., 2000; Winnett et al., 2008) and the prevention of biofilm formation, with no evidence of citrate resistance (Shanks et al., 2006). However, a fatal outcome was associated with citrate administration at 46.7%, causing restrictions on its use in the USA and Canada (Grudzinski et al., 2015).

Safe and effective treated water is the most critical topic for hemodialysis patients, since globally, it is provided almost exclusively by single-pass dialysis systems paired with a reverse osmosis water filtration system (Agar, 2015). Sodium bisulfite has been used to remove chlorine from the feed stream of reverse osmosis systems that operate with polyamide membranes. Our results did not show antifungal activity by the aforementioned chemical agent.

Nowadays, peracetic acid-based disinfectants due to the broad spectrum of microbicidal activity at low concentrations and short exposure times has been used on a large scale (Szewczyk et al., 2013). Peracetic acid breaks down into acetic acid and oxygen, rendering products non-toxic and environmentally safe (Coulliette and Arduino, 2013). Against the *Fusarium* spp. planktonic cells, our results did not show the effectiveness of peracetic acid at 0.1% or 0.2% concentrations, with exposure of 30 and 10 minutes, respectively. In the case of *Aspergillus* spp., there was a reduction of approximately 2 Log_10_ cells/ml after exposure of planktonic cells to 0.1% peracetic acid.

In contrast, Katara et al. (2016) reported the efficiency of peracetic acid (0.1%, 30 min exposure) against *Aspergillus niger* since it reduced the inoculum of 10^8^ cells/ml more than 5Log. A possible explanation for the fact could be the source of the strains tested. While we used strains previously recovered from a hemodialysis facility, probably already adapted to disinfection conditions, Katara et al. (2016) used wild isolates.

The tested concentrations of peracetic acid were effective in reducing 80% of *Fusarium* spp. biofilms cell viability, but only the concentration of 0.2% with a 10-minutes exposure produced the same effect against *Aspergillus* spp. biofilms. Low concentrations of peracetic acid and short exposure times are effective against *Staphylococcus aureus* biofilm (0.5%, 30 seconds of exposure) but not against *Listeria monocytogenes* under the same conditions (Lee et al., 2016). Furthermore, Ilknur et al. (2012) reported that peracetic acid (0.2%) was not completely effective against *Candida* species biofilms (*C. albicans, C. glabrata, C. parapsilosis, C*. *krusei* and *C. tropicalis*) in different times of biofilm formation (6h, 12h, 24h and 48h). Our group previously demonstrated that peracetic acid (3%, 5 min exposure) inhibited the biofilm of *Candida parapsilosis* stricto sensu and *C. orthopsilosis* (Pires et al., 2013).

Together, our results showed that only acetic acid (1%, 30 min) and sodium hypochlorite (2.5%, 30 min) showed fungicidal action against the fungal cells tested. For biofilm cells, in addition to the aforementioned chemical disinfectants, citric acid (50%, 15 min) and peracetic acid (0.1%, 30 min and 0.2%, 10 min) were also effective. This could be explained by the adopted criteria: 99.9% reduction for planktonic cells (Rutala, 1995) and 80% reduction for sessile cells (Van Dijck et al., 2018).

Peracetic acid is mainly used to disinfect dialysis machines during patients’ intervention and dialyzers to allow their reuse (Oliveira et al., 2011). The clinical staff recognized the efficiency of the disinfectant for such purposes. However, a potential toxicity risk is also identified, and residue tests are performed to detect the disinfectant’s presence at the level of ppm (1 ppm). However, two factors have contributed to this monitoring carelessness: the increase in costs for the acquisition of commercial detection systems and the acquisition of more modern dialysis machines with self-disinfection system, especially in the South and Southeast of Brazil. Notably, and unfortunately, when self-disinfecting devices are available, the rinsing time of the machine has been neglected.

In this study, the residual toxicity of peracetic acid was assessed using the histopathological analysis of mice organs exposed to disinfectant and by dosing biochemical and hematological parameters in the same animals. No similar study was found in the literature that would allow us to compare it with the abnormalities found in histology and hematological/biochemical parameters. The few studies with animals (rats and mice) found in the literature, refer to the effects of exposure to vapors and aerosols from lethal and non-lethal doses of commercial preparations of peracetic acid (NRC, 2010).

Merka and Urban (1978) exposed ten mice in a dynamic chamber to aerosols of laboratory peracetic acid in concentrations of 70 to 140 mg/m^3^ for 60 min, three times/week for four weeks, and observed for another two weeks. The animals exposed to peracetic acid showed retarded weight gain compared with controls. The histological examination of the animals revealed lesions in the lungs, which were more accentuated as the disinfectant concentration increased. No injuries were detected in the heart, liver, spleen, or kidneys. In another study (Bock et al., 1975), 30 female ICR Swiss mice (55 to 69 days old) received topical applications of peracetic acid (0.2 ml of 0, 0.3, 1.0, or 3.0% diluted in water) for 5 days/week during 66 weeks. It was reported that 3% of the mice that received 1.0% peracetic acid and 17% of the mice that received 3.0% peracetic acid developed skin cancer. Koch et al. (1989) injected male ICR mice with 0.1% or 0.05% peracetic acid-based disinfectant for 5 consecutive days, reporting abnormalities in the sperm, indicating potential mutagenicity, 36 days after the first injection. Finally, Paldy et al. (1984) showed an increase in mutated chromosomes (17% vs. 3% for controls) in injected mice (intraperitoneal) once daily for 5 days with 1.6 mg of peracetic acid/kg/day.

Similarities such as delayed weight gain, erythrocyte count and decreased hemoglobin concentration when compared to the control group, and lesions in the lungs, kidneys, and liver were observed in animals (rats, mice, calves and pigs) exposed to commercial preparations of peracetic acid, when studied by us or other authors (Benes et al., 1966; Merka and Urban, 1978; Heinze et al., 1979).

## Conclusion

The results showed that despite the variety of disinfectants used in the hemodialysis setting, few of them were shown to be effective against the microorganisms and life forms studied. Against planktonic cells of *Fusarium* spp., fungicidal activity was obtained with 2.5% sodium hypochlorite (30 min exposure) and 1% acetic acid (30 min exposure). However, anti-biofilm activity (80% reduction) was observed for citric acid 50% (15 min exposure), peracetic acid at 0.1% and 0.2% (30 min and 10 min exposure, respectively), and 2.5% sodium hypochlorite (30 min exposure). Against *Aspergillus* spp. planktonic cells, 2.5% sodium hypochlorite (30 min exposure) showed fungicidal action whilst 0.1% peracetic acid (30 min exposure) showed fungistatic activity. For *Aspergillus* spp. biofilm cells, effectiveness was achieved with acetic acid 1% (30 min exposure), citric acid 50% (15 min exposure), peracetic acid 0.2% (10 min exposure), and 2.5% sodium hypochlorite (30 min exposure). In residual toxicity assays, mice exposed to 1 ppm peracetic acid for 60 weeks resulted in liver atrophy. Anatomopathological analyzes of internal murine organs showed changes in the architecture of the liver, lungs, spleen, and pancreas and minor necrotic and congestive changes in the lungs and spleen. Biochemical determinations revealed normal ALT, AST, and albumin values, although compared to the control group, the animals exposed to the disinfectant showed significantly higher ALT values. The hematological parameters included quantifying red blood cells, hemoglobin, hematocrit, white blood cells, and platelets, which remained normal. However, the comparison with the control group showed significant differences with the group exposed to the disinfectant in terms of red cells and hematocrit, which were lower. Platelets were also lower than the control group, but not significantly. Adjusting the guidelines regarding the organisms’ identification and microbial control can offer more safety to patients dependent on hemodialysis treatment, providing more remarkable survival and better quality of life.

## Data Availability Statement

The original contributions presented in the study are included in the article/supplementary material. Further inquiries can be directed to the corresponding author.

## Ethics Statement

The animal study was reviewed and approved by Ethics Committee on the Use of Animals in research at the University of Franca.

## Author Contributions

LGL, LAC, JC, CT, and LOL contributed to the investigation, the methodology, and the writing of the original draft. RF, AO, SA-J, and LZC performed the animal analysis. RP, MM, and LGL contributed to the partial funding acquisition. RP, MG, and MM contributed to the conceptualization, the methodology, the scientific advice, the project administration, and the review and editing of the manuscript. All authors contributed to the article and approved the submitted version.

## Funding

This study was partially funded by the São Paulo Research Foundation (FAPESP), grants 2015/19090-5, and Coordination for the Improvement of Higher Education Personnel – Brazil (CAPES) Finance Code 001.

## Conflict of Interest

The authors declare that the research was conducted in the absence of any commercial or financial relationships that could be construed as a potential conflict of interest.
